# Temporal Relation Modeling and Multimodal Adversarial Alignment Network for Pilot Workload Evaluation

**DOI:** 10.1109/JTEHM.2025.3542408

**Published:** 2025-02-14

**Authors:** Xinhui Li, Ao Li, Wenyu Fu, Xun Song, Fan Li, Qiang Ma, Yong Peng, Zhao LV

**Affiliations:** Anhui Province Key Laboratory of Multimodal Cognitive Computation, School of Computer Science and TechnologyAnhui University 12487 Hefei 230601 China; CAAC Key Laboratory of Civil Aviation Flight Technology and Flight SafetyCivil Aviation Flight University of China 117786 Guanghan 618307 China; Mianyang Flight CollegeCivil Aviation Flight University of China 117786 Mianyang 621000 China; School of Computer Science and TechnologyHangzhou Dianzi University 12626 Hangzhou 310018 China; Key Laboratory of Flight Techniques and Flight Safety, CAAC Beijing 100710 China

**Keywords:** Pilot workload evaluation, transformer, adversarial alignment, electroencephalogram, electromyography

## Abstract

Pilots face complex working environments during flight missions, which can easily lead to excessive workload and affect flight safety. Physiological signals are commonly used to evaluate a pilot’s workload because they are objective and can directly reflect physiological mental states. However, existing methods have shortcomings in temporal modeling, making it challenging to fully capture the dynamic characteristics of physiological signals over time. Moreover, fusing features of data from different modalities is also difficult.To address these problems, we proposed a temporal relation modeling and multimodal adversarial alignment network (TRM-MAAN) for pilot workload evaluation. Specifically, a Transformer-based temporal relationship modeling module was used to learn complex temporal relationships for better feature extraction. In addition, an adversarial alignment-based multi-modal fusion module was applied to capture and integrate multi-modal information, reducing distribution shifts between different modalities. The performance of the proposed TRM-MAAN method was evaluated via experiments of classifying three workload states using electroencephalogram (EEG) and electromyography (EMG) recordings of eight healthy pilots.Experimental results showed that the classification accuracy and F1 score of the proposed method were significantly better than the baseline model across different subjects, with an average recognition accuracy of $91.90~\pm ~1.72\%$ and an F1 score of $91.86~\pm ~1.75\%$.This work provides essential technical support for improving the accuracy and robustness of pilot workload evaluation and introduces a promising way for enhancing flight safety, offering broad application prospects. Clinical and Translational Impact Statement: The proposed scheme provides a promising solution for workload evaluation based on electrophysiological signals, with potential applications in aiding the clinical monitoring of fatigue, mental status, cognitive psychology, and other disorders.

## I. Introduction

Pilots’ flight workload levels have significant impact on aviation safety [1]. The various psychological and physiological stresses experienced by pilots during flight missions can lead to varying degrees of workload, and workload imbalance is one of the significant threats to flight safety [2]. Recent statistical data indicate that 75% of safety accidents in civil aviation transportation are related to human factors, with the majority resulting from crew errors [3], [4]. Therefore, a comprehensive evaluation of pilots’ operational behavior, psychological, and physiological state during flight tasks, especially workload levels, can help pilots perform their tasks safely and efficiently.

Existing evaluation methods can be divided into two categories, subjective and objective evaluation methods. Subjective evaluation methods rely on the flight instructor’s observation of the pilots’ behavioral performance or adopt evaluation scales filled out by the pilot [5], [6], such as the NASA Task Load Index (NASA-TLX) scale [7] and the Subjective Workload Assessment Technique (SWAT) scale [8]. Although these methods are simple and intuitive, they can be easily affected by biased individual observation and experience, and it is also challenging to reflect the workload status in real-time. In contrast, the objective evaluation methods adopt statistical models by measuring the pilots’ physiological signals, flight parameters, and other information [9], [10]. These methods can objectively reflect the pilots’ cognitive and physiological state, reducing the interference of subjective factors and improving the evaluation accuracy. Among them, the flight parameters can reflect the pilots’ operational behavior and the status of onboard equipment during missions. Still, they cannot directly reflect their psychological and physiological status, limiting their ability to evaluate the pilots’ workload comprehensively [11]. In contrast, physiological signals such as electroencephalogram (EEG), electromyography (EMG), and heart rate variability (HRV) can reflect the real-time physiological status of pilots directly and accurately [12], [13]. For example, Zhu et al. [14] collected photoplethysmography (PPG) signals during three stages of helicopter flight and extracted their HRV features, which were classified by three commonly employed machine learning algorithms, ultimately achieving a classification accuracy of 85.9%. Mohanavelu et al. [15] extracted spectral features from EEG signals and conducted research on brain source localization using low-resolution brain electromagnetic tomography (LORETA). They identified brain source locations related to pilot cognitive workload by examining the neurodynamic responses to specific tasks. In this paper, we focus on physiological signals based workload evaluation.

Despite the success of traditional workload evaluation methods, they typically recognize the pilots’ workload status by combining manual feature extraction methods with machine-learning-based classifiers. For instance, Mohanavelu et al. [16] manually extracted HRV and EEG features to evaluate low and high workloads across three stages of flight experiments, employing three machine learning classification algorithms (Linear Discriminant Analysis (LDA), k-Nearest Neighbors (k-NN), and Support Vector Machine (SVM)) to classify workload conditions. Jun et al. [17] proposed a sensitivity analysis method based on comprehensive testing and developed a SVM-based real-time assessment model for the pilot workload. Despite the promising results of these methods, traditional machine learning methods rely heavily on the quality of feature extraction and face challenges in handling high-dimensional features, making it difficult to uncover task-related features. Additionally, manual feature extraction depends significantly on the researchers’ prior knowledge and is closely tied to specific task data and scenarios, resulting in poor adaptability [18], [19].

To overcome these limitations, deep learning techniques have been proposed for their powerful representation learning capabilities. They can capture complex relationships between features and reduce the dependence on manual feature extraction. For instance, Lee et al. [20] fed EEG signals collected from pilots into a multiple-feature block-based convolutional neural network (MFB-CNN) with spatiotemporal EEG filters to classify four psychological states of pilots. Similarly, Deng et al. [21] designed a Stacked Shrinkage Sparse Autoencoder (CSAE) network, which enhanced the ability to discover and extract local EEG features, thereby improving the performance of pilot workload evaluation. By using CNN and LSTM for classification, these methods have achieved competitive performance. Both network architectures have their unique advantages. Precisely, CNN can effectively extract local features and is well-suited for processing the spatial structure of image and sequence data [22]. LSTM can capture temporal dependencies in sequence data, making them ideal for processing time series data [23].

Furthermore, considering the limitations of a single modality and the complementarity of different modalities, multimodal physiological signals have been utilized and proven to be effective for improving evaluation accuracy compared to a single modality [24], [25]. For example, Han et al. [26] collected various physiological signals, including EEG, and used a multimodal deep learning (MDL) network containing LSTM and CNN to classify different psychological states, significantly improving the accuracy of pilot psychological state classification. Similarly, Yu et al. [27] proposed a multimodal convolutional bidirectional LSTM network (MCBLN) to fuse the spatiotemporal correlation features of EEG signals and peripheral physiological signals collected through experiments, aiming to detect the drowsiness level of pilots.

By summarizing existing work, we observe that modeling temporal relationships and multimodal fusion are crucial. In temporal relationship modeling, current approaches primarily use CNNs or LSTMs, which cannot handle long-distance dependencies or suffer from low efficiency. In multimodal fusion, existing methods often overlook the differences between different modalities and employ simple fusion strategies, leading to suboptimal performance. For example, the recent work Husformer [28] performed multimodal fusion by using the cross-attention mechanism in the Transformer, without considering aligning features from different modalities before feature fusion. Moreover, it concatenated features from different modalities along the temporal dimension to perform self-attention for context information aggregation, which incurred significant computation costs due to the quadratic complexity of the self-attention operation.

Based on the above discussion, we proposed a novel Temporal Relation Modeling and Multimodal Adversarial Alignment Network (TRM-MAAN) for Pilot Workload Evaluation, which mainly included a temporal modeling module based on Transformer [29] and a multimodal (EEG and EMG) fusion module based on adversarial alignment. In the temporal modeling module of TRM-MAAN, Transformer block was used as multi-modality feature extraction module, utilizing their self-attention mechanism to capture the global relationships between elements in the sequence. Through their self-attention mechanism, Transformers can provide robust feature extraction and fusion capabilities [30], [31], [32], effectively addressing the shortcomings of CNNs and LSTMs. In the adversarial alignment-based multimodal fusion module, we captured and integrated multimodal information more effectively through mutual adversarial training between modality classifier and multi-modality feature fusion module, thereby reducing distribution shifts between different modalities and achieving effective alignment. By combining these two designs, the proposed TRM-MAAN can effectively model temporal relationships and align multimodal features, providing a comprehensive framework for pilot workload evaluation. This work contributes to evaluating and monitoring pilots’ workload status, which is essential for safeguarding pilots’ healthcare. In addition, the proposed method has potential value for clinically monitoring fatigue, psychological state, cognitive psychology, and other disorders. The contributions of this paper can be summarized as follows:

• A novel pilots’ workload evaluation method that combines the multimodal physiological signals of electroencephalography (EEG) and electromyography (EMG) is proposed to achieve end-to-end workload evaluation.
• A temporal relation modeling module based on Transformer is designed, which is capable of capturing longtime dependencies and handling high-dimensional features, thus enabling the model to learn complex spatiotemporal relationships more efficiently.
• The multimodal adversarial alignment mechanism is applied for the first time to the workload evaluation of pilots. This mechanism reduces the distribution shifts between different modalities and achieves effective alignment, thus improving the robustness and reliability of the proposed method.

The rest of this paper is organized as follows. Section II describes the dataset collected from subjects conducting simulated flights and TRM-MAAN. Section III introduces the performance evaluation of our proposed method. Section IV discusses the experimental results, and the final section presents the conclusion.

## II. Material and Method

**FIGURE 1.**
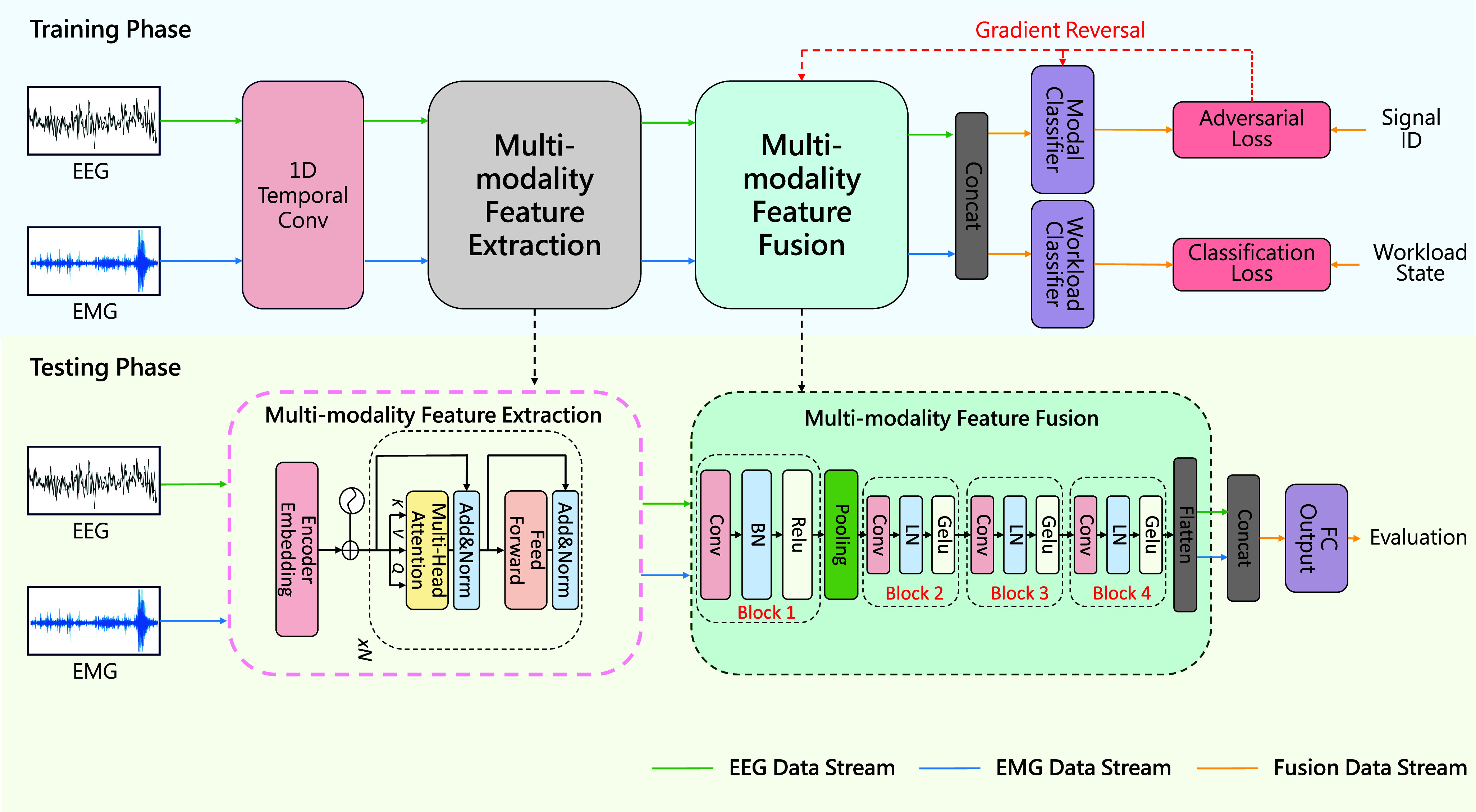
The model structure of TRM-MAAN. The blue arrows indicate the flow of EEG data, the green arrows indicate the flow of EMG data, the dashed arrows denote the gradient reversal operation, and the orange arrows show the flow of the fused multimodal data.

Fig. 1 illustrates the flowchart of TRM-MAAN. Initially, EEG and EMG signals generated by pilots during different flight stages were collected simultaneously. Both signals were segmented into a series of time windows and then fed into the dual-branch network of the model simultaneously. For each EEG and EMG sample in one window, features were extracted using one-dimensional temporal convolution and Transformer models. These features were then aligned through the adversarial training and fed into the classification module, obtaining the predicted pilot workload.

### A. Subjects and Experiments

Eight male pilots (S1-S8, age: $22~\pm ~0.60$ years; body mass: $72~\pm ~1.30$ kg; height: $180~\pm ~2.00$ cm; mean ± standard deviation) were recruited in this study. All pilots who did not have any neuromuscular diseases were informed of the experimental procedures and signed the informed consent. The study was approved by the Ethics Review Board of the Anhui University (No. BECAHU-2024-003, March 18, 2024, Hefei, China).

**FIGURE 2.**
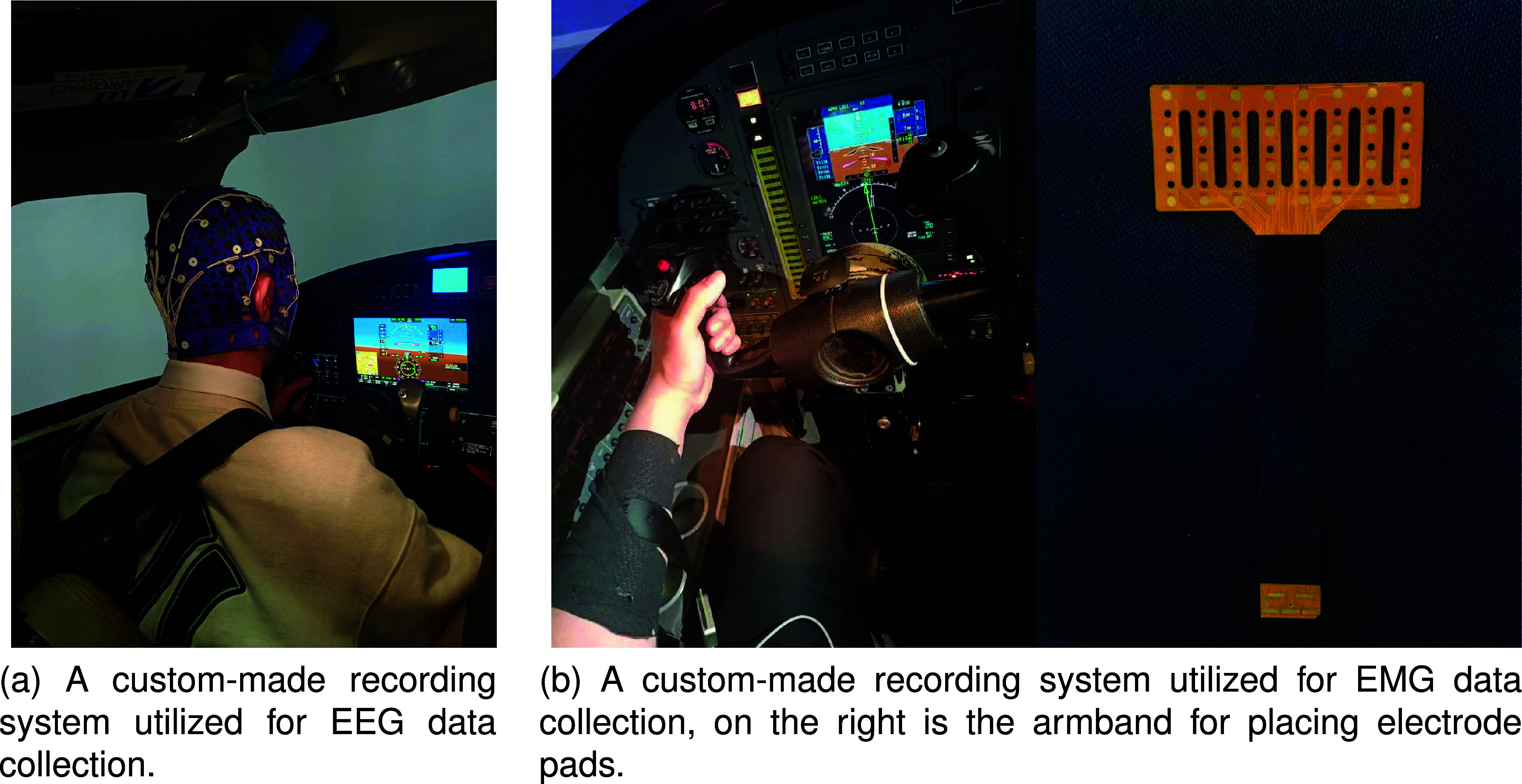
The diagram of the experiment set-up.

The EEG data was recorded at a sampling rate of 2kHz using an EEG system (Compumedics Grael, Compumedics, Australia). The layout of the 32-channel EEG electrodes on the cap follows the international 10-20 system, as shown in Fig. 2a. Curry 8 (Compumedics, Australia) analysis software was used for data analysis. At the same time, EMG data were recorded dominantly from the extensor digitorum muscles of all pilots. The recording was conducted on the left hand while the pilots maneuvered the flight stick corresponding to the main function of the tested muscles. As shown in Fig. 2b, a 32-channel unipolar electrode array was used, arranged in a $4\times 8$ grid, with each electrode having a diameter of 2 mm and an electrode spacing of 4 mm. A multichannel surface EMG data recording system (FlexMatrix Inc., Shanghai, China) was used for data recording. It consisted of a two-stage amplifier with a total gain of 60 dB, a bandpass filter set at 1- 500 Hz for each channel, and an analog-to-digital converter (RHD 2132, Intan, Inc.) with a sampling rate of 1 kHz. All recorded EEG and EMG data were stored in the hard drive of a laptop computer and subsequently processed by MATLAB software (version R2018b, MathWorks Natick, USA).

To eliminate the effects of external factors, we asked all pilots to perform the same tasks at the same time of day. In addition, we endeavored to ensure that the pilots maintained a consistent state before the start of the experiment. Specifically, we asked pilots to maintain regular sleep, refrain from caffeine intake, and avoid flying missions for three days before the experiment. Before the start of the experiment, pilots were asked to complete a self-report questionnaire to ensure that all pilots were in a relaxed state both physically and psychologically. At the beginning of the experiment, the subject’s skin was first cleaned with medical alcohol, and a conductive paste was applied to the head and arm electrode placements. Then, the EEG and EMG electrode arrays were placed at the corresponding locations. As shown in Fig. 2b, the EMG electrode arrays were placed using a retractable armband to secure the electrode arrays to the subject’s arm to prevent dislodgement. All pilots were asked to complete a flight mission in a dynamic simulator for 35-40 minutes. The mission consisted of five phases, as described in detail in Table 1.

**TABLE 1:** The Flight Stage and Specific Missions

Stage Number	Stage Name	Mission
No.1	Single-Engine Departure	Turn off one engine to accelerate the aircraft to takeoff speed, lift off the ground, and control the plane to climb steadily to the designated altitude and route.
No.2	Waiting	Maintain the aircraft at the predetermined altitude and speed, calculate and adjust fuel consumption, ensure flight safety, and maintain communication with the ground control tower while awaiting further instructions.
No.3	Approach	The transition from continuous waiting to interception.
No.4	Interception	Confirm the direction and position of the runway, align the aircraft with the runway, and control the plane to gradually lower its altitude along the approach path, preparing for landing.
No.5	Landing Roll	Control the safe landing of the aircraft, decelerate to taxiing speed, guide the plane along the designated taxiway, enter the taxiway, and finally stop the aircraft at the designated parking spot to complete the flight mission.

We used the NASA-TLX scale to measure the workload levels, which is the most commonly used method internationally when evaluating pilot workload [14], [33]. The NASA-TLX scale was filled in immediately after each pilot completed the flight task, and the scale scores were classified into three levels according to the threshold selection method [14].

### B. Signal Pre-Processing

A 0.1-30 Hz bandpass filter and a 75-500Hz were applied to EEG and EMG signals respectively to eliminate noise artifacts. The filtered EEG and EMG signals were split into several non-overlapping time windows with a window length of 1 second. Since the EEG and EMG signals have different sampling rates (2kHz for EEG and 1kHz for EMG), we downsampled the EEG data in the 1s signal window twice, corresponding to data points 1, 3, 5..., and 2, 4, 6..., respectively, to create two sets of EEG data with an equal number of sample points as the 1-second EMG signal. Downsampling of the EEG is commonly used and effective [34], [35]. Finally, these downsampled EEG signals were individually paired with the EMG signals, and the paired samples were considered as basis samples for further classification tasks.

### C. Model Structure Overview

The model structure of TRM-MAAN consists of four parts, multi-modality feature extraction module *E*, multi-modality feature fusion module *F*, modal classifier *M*, and workload classifier *C*. multi-modality feature extraction module *E* is a Transformer architecture consisting of two parts: an Encoder and a Decoder, relying on a Multi-Head Self-Attention mechanism to capture global contextual information in the input sequence. The multi-modality feature fusion module *F* is a multi-layer architecture consisting of four consecutive blocks, a max pooling layer, and a flatten layer. The number of blocks is usually proportional to the amount of data, and in this work, it was determined through preliminary experiments. Each block consists of a convolutional layer, a normalization layer, and an activation layer. Specifically, the first block is a custom structure that includes a convolutional layer with a kernel size of 3, a batch normalization layer (BN layer), and a ReLU activation layer. The following three blocks use the first three blocks of the ConvNeXt network, each block consists of a convolutional layer with a kernel size of 7, a layer normalization layer, and a Gelu layer. The max pooling layer is located between the first and second blocks, with a filter size of $2\times 2$ and a stride of 2. The last layer is the flatten layer, which transforms the three-dimensional representation into a one-dimensional representation, containing learning features of different modalities that can be used for classification. Additionally, the modal classifier *M* consists of two fully connected (FC) layers responsible for classifying modal IDs, while the workload classifier *C* includes two FC layers for evaluating the pilots’ workload. It is worth noting that the modal classifier *M* is only used during the training phase to learn standard features between modalities and improve the model’s generalization ability.

### D. Model Training

Before the training phase, the paired samples in the training set were augmented using random flipping and adding Gaussian noise, which has been shown effective for improving the robustness to noise interference in real-world data [36], [37]:

\begin{align*} & X'_{\left \{{{ e, m }}\right \}} = filp(X_{\left \{{{ e, m }}\right \}}) \\ & X"_{\left \{{{ e, m }}\right \}} = X_{\left \{{{ e, m }}\right \}} + \mathcal N\left ({{ 0, \sigma ^{2} }}\right) \\ & \hat {X}_{\left \{{{ e, m }}\right \}} = \left \{{{ X'_{\left \{{{ e, m }}\right \}}, X"_{\left \{{{ e, m }}\right \}} }}\right \}, \tag {1}\end{align*}

where $X_{e}$ is EEG signal and $X_{m}$ is EMG signal. One-dimensional temporal convolution was used to project them into the same dimension *D* for a preliminary temporal transformation:

\begin{equation*} \dot {X}_{\left \{{{ e, m }}\right \}} = Conv1D(\hat {X}_{\left \{{{ e, m }}\right \}}, k_{\left \{{{ e, m }}\right \}}), \tag {2}\end{equation*}

where $\dot {X}$ denotes a convolutional sequence with the same dimension *D*. $k_{\left \{{{ e, m }}\right \}}$ represents the time convolution kernel size for EEG and EMG. Then, traditional sine and cosine functions were used to encode $\dot {X}$ to obtain feature $O_{\left \{{{ e, m }}\right \}}$ containing positional information, which was input into the attention layer of the Transformer:

\begin{equation*} O_{\left \{{{ e, m }}\right \}} = \dot {X}_{\left \{{{ e, m }}\right \}} + PE_{\dot {X}_{\left \{{{ e, m }}\right \}}}, \tag {3}\end{equation*}

where $PE_{\dot {X}_{\left \{{{ e, m }}\right \}}}$ represents the positional information obtained by encoding the convolutional sequence $\dot {X} \in \mathbb {R}^{L, D}$, which can be defined as a matrix:

\begin{align*} PE_{\dot {X}}\left [{{ pos, 2k }}\right ] = \sin \left ({{ \frac {pos}{10000^{\frac {2k}{D}}} }}\right) \\ PE_{\dot {X}}\left [{{ pos, 2k+1 }}\right ] = \cos \left ({{ \frac {pos}{10000^{\frac {2k}{D}}} }}\right), \tag {4}\end{align*}

where *L* represents the length of the convolutional sequence, *pos* denotes the position of the token in the sequence, with $pos\in \left [{{ 1, \ldots, L }}\right]$, and $k\in \left [{{ 1, \ldots, \frac {D}{2} }}\right )$.

In addition to positional information, temporal relation modeling is crucial for improving features’ robustness and smoothness to correctly classifying samples. Therefore, the output $O_{\left \{{{ e, m }}\right \}}$ was then fed into the multi-head attention module to aggregate temporal information. Specifically, the input sequence vector $O_{\left \{{{ e, m }}\right \}}$ was multiplied with three matrices to obtain query *Q*, key *K*, and value *V*:

\begin{align*} Q & = O_{\left \{{{ e, m }}\right \}}\cdot W_{Q} \\ K & = O_{\left \{{{ e, m }}\right \}}\cdot W_{K} \\ V & = O_{\left \{{{ e, m }}\right \}}\cdot W_{V}, \tag {5}\end{align*}

where the matrices $W_{Q}\in \mathbb {R}^{D, D_{Q}}$, $W_{K}\in \mathbb {R}^{D, D_{K}}$, and $W_{V}\in \mathbb {R}^{D, D_{V}}$ are learnable weight parameters. Using these, the output of the self-attention layer can be obtained as follows:

\begin{equation*} O_{\left \{{{ e, m }}\right \}}^{head_{i}} = softmax\left ({{ \frac {Q\cdot K^{T}}{\sqrt {D_{K}}} }}\right) \cdot V, \tag {6}\end{equation*}

and then the output of the multi-head self-attention layer can be defined as:

\begin{equation*} O_{\left \{{{ e, m }}\right \}}^{Mul} = concat\left ({{ O_{\left \{{{ e, m }}\right \}}^{head_{1}}, \ldots, O_{\left \{{{ e, m }}\right \}}^{head_{n}} }}\right), \tag {7}\end{equation*}

where *n* is the head number. The output of each multi-head self-attention layer was further processed and transformed into features through a feedforward network, which also includes residual connections and layer normalization to improve efficiency and stability. The final features extracted by multi-modality feature extraction module *E* can be represented as:

\begin{equation*} Z_{\left \{{{ e, m }}\right \}} = LayerNorm\left ({{ O_{\left \{{{ e, m }}\right \}}^{Mul} + FeedForward\left ({{ O_{\left \{{{ e, m }}\right \}}^{Mul} }}\right) }}\right). \tag {8}\end{equation*}

With the output features of each sample $Z_{\left \{{{ e, m }}\right \}}$, the multi-modality feature fusion module *F* can extract highly correlated features with the load state, while the modal classifier $M^{cls}$ and the workload classifier $C^{cls}$ respectively obtain the modal classification result $\hat {Y}^{M}$ and the workload evaluation result $\hat {Y}^{C}$:

\begin{align*} \hat {Y}^{M} & = M^{cls}\left ({{ F\left ({{ Z_{\left \{{{ e, m }}\right \}} }}\right) }}\right), \\ \hat {Y}^{C} & = C^{cls}\left ({{ Concat\left ({{ F\left ({{ Z_{e} }}\right), F\left ({{ Z_{m} }}\right)}}\right) }}\right). \tag {9}\end{align*}

In the training process, adversarial loss and classification loss can be calculated based on the results of $\hat {Y}^{M}$ and $\hat {Y}^{C}$. Specifically, adversarial loss can be expressed as:

\begin{equation*} L_{adv} = -\sum _{i=1}^{K}y_{i}log\left ({{ \hat {Y}^{M} }}\right), \tag {10}\end{equation*}

where $y_{i}$ is the actual label of the modal ID, and *K* represents the number of modalities, which is 2 in this work.

During the adversarial loss training process, the modal classifier *M* was committed to correctly classifying modal IDs, and its optimization for adversarial loss can be expressed as $min_{M}L_{adv}$. Conversely, the multi-modality feature fusion module *F* strived to prevent *M* from accurately classifying modal IDs by optimizing its parameters, and the optimization of adversarial loss can be expressed as $max_{F}L_{adv}$. Additionally, based on the final unimodal features of the concatenated EEG and EMG, the calculated workload classification loss $L_{cls}$ can be expressed as:

\begin{equation*} L_{cls} = -\sum _{i=1}^{N}c_{i}log\left ({{ \hat {Y}^{C} }}\right), \tag {11}\end{equation*}

where $c_{i}$ represents the actual label of the workload state, *N* denotes the number of workload state categories (i.e., $N =3$ in this work).

Additionally, the inconsistent optimization directions of the modal classifier *M* and the multi-modality feature fusion module *F* complicate the optimization of deep neural networks using standard gradient descent. To address this, we employ a gradient reversal operation to invert the gradient values of the multi-modality feature fusion module generated by adversarial loss. This allows our model to learn in an end-to-end way by minimizing the total loss *L* in Equation (12).

\begin{equation*} L = L_{cls} + L_{adv}. \tag {12}\end{equation*}

For the hyper-parameter settings, the learning rate was set to $10^{-3}$, and gradients with absolute values exceeding ten were clipped to stabilize the training process.

### E. Model Testing and Decision Making

In the testing phase, given the testing EEG and EMG data $X_{e}^{t}, X_{m}^{t} \in \mathbb {R}^{L, D}$, we feed it into the proposed method to obtain the workload classification result $\hat {Y}^{C} \in \mathbb {R}^{N}$. Since $\hat {Y}^{C}$ represents a probability distribution, the predicted workload status label *c* can be determined as follows:

\begin{equation*} c = argmax_{i}\hat {Y}^{C}(i). \tag {13}\end{equation*}

### F. Performance Evaluation

To comprehensively verify the proposed method’s performance, the training set and testing set were divided in the proportion of 8:2 for each subject’s data. We conducted a five-fold cross-validation of each participant’s data. Specifically, the dataset was randomly divided into five parts: four were used for training and the remaining for testing. This process was repeated five times, each part of the data serving as the testing set once. We used accuracy (*Acc*) and multi-class average F1 score ( $F1$) as the main evaluation metrics. *Acc* measures the proportion of correctly classified samples in the testing set relative to the total number of samples, while $F1$ describes the model’s ability to capture positive samples. The corresponding formulas for *Acc* and $F1$ are as follows:

\begin{align*} Acc & = \frac {TP + TN}{TP + TN + FP + FN}, \tag {14}\\ F1 & = 2 \cdot \frac {\frac {TP}{TP + FP}\cdot \frac {TP}{TP + FN}}{\frac {TP}{TP + FP} + \frac {TP}{TP + FN}}, \tag {15}\end{align*}

where *TP* (True Positive) represents the number of samples correctly classified as positive, *TN* (True Negative) represents the number of samples correctly classified as unfavorable, *FP* (False Positive) represents the number of samples incorrectly classified as positive, and *FN* (False Negative) represents the number of samples incorrectly classified as unfavorable.

To validate the superiority of TRM-MAAN, we compared it with two multimodal-based methods previously studied for emotion recognition which is similar to pilot workload evaluation: MhyEEG and Husformer. In addition, we also compared the effectiveness of TRM-MAAN with the classical method EEGNet. The details of the compared methods are described below:

• EEGNet [38]: EEGNet is a lightweight convolutional neural network composed of a regular convolutional layer, a depthwise convolutional layer, and a detachable convolutional layer. It is specifically designed for processing EEG signals. Due to its simple and efficient structure, EEGNet is widely used in various EEG signal classification tasks.
• MHyEEG [39]: MHyEEG is an advanced multimodal network featuring a novel fusion module defined in the hypercomplex domain. It consists of two main components, an encoder and a hypercomplex fusion module. The encoder processes each modality independently, producing unimodal combined features for the hypercomplex fusion module. This fusion module can capture global and local relationships between feature dimensions, effectively leveraging the correlations present in different physiological signals to learn more robust representations.
• Husformer [28]: Husformer is an end-to-end multimodal Transformer framework that employs a cross-modal Transformer to focus on the potential correlations revealed by all modalities. It then utilizes a self-attention Transformer to further prioritize contextual information within the fused representation, thereby effectively integrating multiple physiological signals.

Additionally, we designed ablation experiments to verify the importance of each component in the model. The specific details of the ablation experiments were shown in Table 2. To verify the effectiveness of adversarial alignment, we designed the TRM method, in which the features of both modalities were fused using concatenation. We also employed the MAAN method to validate the effectiveness of the Transformer-based multi-modality feature extraction, where the features of the two modalities were extracted only by one-dimensional temporal convolution and then fed into the multi-modality feature fusion module. Finally, to compare the ability of the proposed methods to characterize unimodal signals, only EEG and EMG were used as input signals in the design of the Uni-EEG and Uni-EMG methods, respectively, and a comparative model was used for experiments using EEGNet with the unimodal input signal.

**TABLE 2:** Details of Ablation Methods

Method	Adversarial Alignment	Transformer	EEG	EMG
TRM	$\times $	✓	✓	✓
MAAN	✓	$\times $	✓	✓
Uni-EEG	$\times $	✓	✓	$\times $
Uni-EMG	$\times $	✓	$\times $	✓

A one-way repeated-measure ANOVA was applied to the classification accuracy of the proposed method versus the contrast methods mentioned above. The significance level was set as $p < 0.05$. All statistical analysis were implemented by SPSS software (version 24.0, SPSS Inc. Chicago, IL, USA). All training and experiments were conducted on an NVIDIA Tesla A100 GPU, and the code was implemented using the Pytorch library.

## III. Result

**FIGURE 3.**
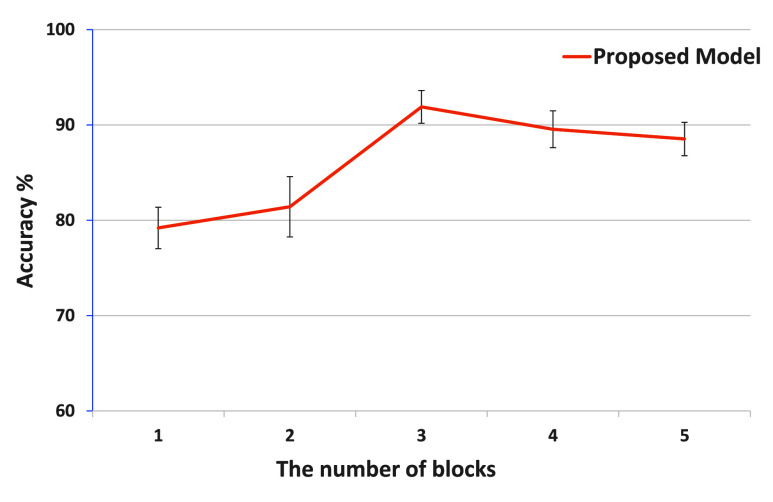
The classification accuracy averaged over all subjects using different numbers of blocks with TRM-MAAN, Error bars represent the standard deviations.

Fig. 3 illustrates the impact of block numbers on classification accuracy. It was shown that when the number of blocks was set to 3, the model achieved optimal performance with an average accuracy of $91.90~\pm ~1.72\%$ ( $p < 0.05$). Increasing or decreasing the block number resulted in a slight reduction in classification accuracy. Therefore, this work fixed the number of blocks at three and applied this configuration in subsequent analyses.

Table 3 shows the *Acc* and $F1$ of TRM-MAAN across all subjects. It can be observed that the average accuracy of TRM-MAAN on all subjects was $91.90~\pm ~1.72\%$. Specifically, TRM-MAAN achieved the best performance on subject 4, with an *Acc* of $93.94~\pm ~1.05\%$ and an $F1$ of $93.95~\pm ~0.99\%$. In contrast, the model’s performance on subject 8 was the most miniature ideal, but the *Acc* and $F1$ still reached $89.01~\pm ~1.07\%$ and $88.98~\pm ~1.08\%$, indicating the competitiveness of the proposed method.

**TABLE 3:** The Average and Standard Deviation (%) of ACC and $F1$ for the TRM-MAAN Across all Subjects

Subject	Acc (Mean ± Std)	F1 (Mean ± Std)
S01	$90.52~\pm ~1.37$	$90.26~\pm ~1.49$
S02	$94.37~\pm ~1.08$	$94.32~\pm ~1.15$
S03	$92.23~\pm ~1.38$	$92.21~\pm ~1.39$
S04	$93.94~\pm ~1.05$	$93.95~\pm ~0.99$
S05	$90.89~\pm ~0.42$	$90.87~\pm ~0.51$
S06	$93.14~\pm ~0.33$	$93.15~\pm ~0.32$
S07	$91.13~\pm ~1.38$	$91.11~\pm ~1.40$
S08	$89.01~\pm ~1.07$	$88.98~\pm ~1.08$
Average	$91.90~\pm ~1.72$	$91.86~\pm ~1.75$

**FIGURE 4.**
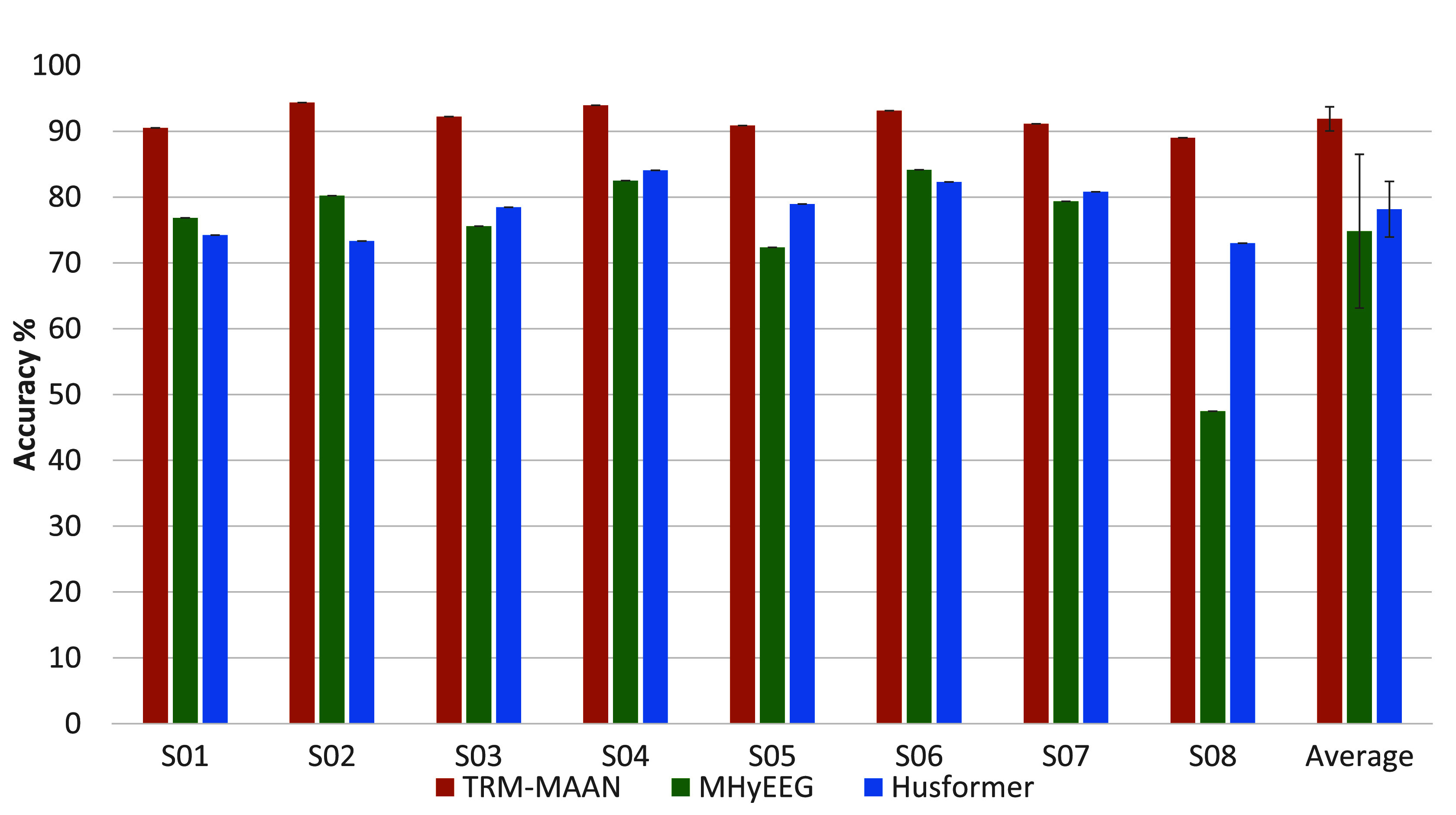
The *ACC* of workload evaluation for all subjects using the TRM-MAAN and two comparative methods.

Fig. 4 shows the classification accuracies across subjects when using TRM-MAAN and the two multimodal baseline models (MHyEEG, Husformer), where the right panel displays the average accuracy of all subjects. The results show that the proposed TRM-MAAN method achieved an average classification accuracy of $91.90~\pm ~1.72\%$, significantly better than the other two models, with statistical significance ( $p < 0.05$). The average classification accuracy of MHyEEG and Husformer models were $74.81~\pm ~10.68\%$ and $78.14~\pm ~4.23\%$, respectively, which were 17% and 13% lower than our model. Table 4 shows the performance of the three methods on different metrics, and it can be seen that TRM-MAAN achieved optimal results on all metrics with statistical significance ( $p < 0.05$).

**TABLE 4:** Performance Metrics of TRM-MAAN and Comparative Models on the Dataset. Note That Values Were Presented as Mean ± Standard Deviation Across all Test Samples

Method	Accuracy	F1 Score	Precision	Recall	AUC
TRM-MAAN	$91.90~\pm ~1.72$	$91.86~\pm ~1.75$	$91.92~\pm ~2.52$	$90.88~\pm ~3.41$	$95.32~\pm ~2.15$
MhyEEG	$74.81~\pm ~10.68$	$74.14~\pm ~11.32$	$75.03~\pm ~8.56$	$73.12~\pm ~12.45$	$76.89~\pm ~9.34$
Husformer	$78.14~\pm ~4.23$	$77.91~\pm ~3.98$	$78.45~\pm ~5.01$	$77.33~\pm ~4.89$	$79.21~\pm ~3.87$

**FIGURE 5.**
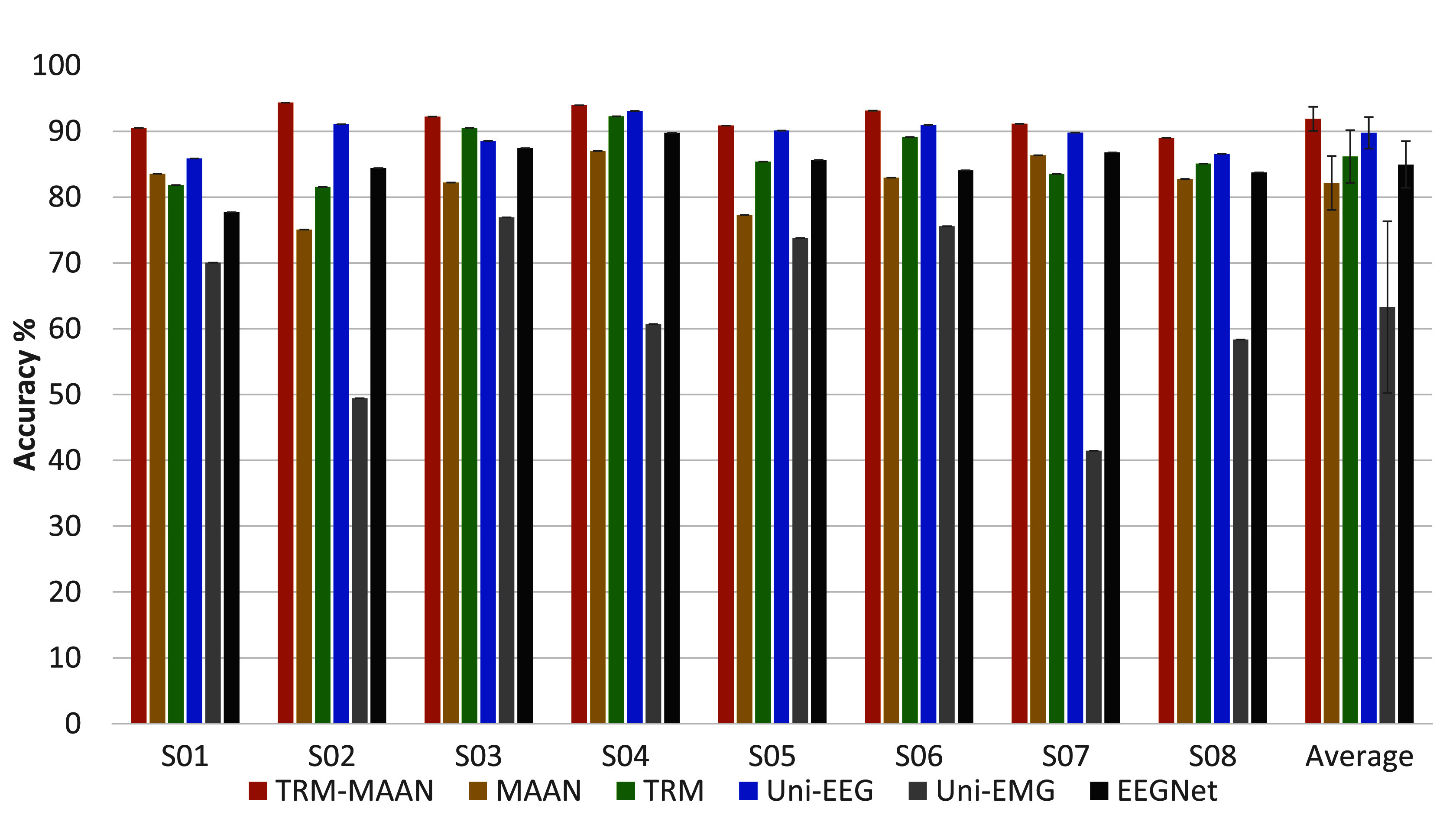
The *ACC* of workload evaluation for all subjects using the TRM-MAAN and ablation methods.

Additionally, ablation experiments were conducted on all subjects using TRM-MAAN to quantify each component’s contributions. As shown in Fig. 5, after removing the adversarial alignment mechanism, the average accuracy on all subjects decreased by 9.76% to $82.14~\pm ~4.10\%$. Similarly, after the Transformer was removed, the average accuracy on all subjects decreased by 5.75% to $86.15~\pm ~4.03\%$.

To further validate the real-time performance of TRM-MAAN, we calculated the average deployment time (i.e., time delay) and computational complexity, i.e., GPU memory usage (Mem) and Multiply-Accumulate Operations (MACs) for each sample. The time delay of each sample window was the sum of the downsampling preprocessing time and the network inference time. In this study, the average downsampling time for the sample window was $5.1286\times 10^{-3}$ seconds. Table 5 lists the average time delay, Mem, and MACs per sample when using different models. It can be found that the TRM-MAAN achieved the lowest time delay of $12.7672\times 10^{-3}$ Sec, lowest Mem of 374.0752 MB, and lowest MACs of 180.425 MB.

**TABLE 5:** The Average Time Delay, GPU Memory Usage, and Multiply-Accumulate Operations (MACs) Per Sample Using Different Models in the Testing Phase

Method	Time delay	Mem	MACs
TRM-MAAN	$12.7672\times 10^{-3}$ Sec	374.0752 MB	180.425 MB
MhyEEG	$13.6925\times 10^{-3}$ Sec	662.4944 MB	204.612 MB
Husformer	$16.9125\times 10^{-3}$ Sec	5,999.8252 MB	235.409 MB

**FIGURE 6.**
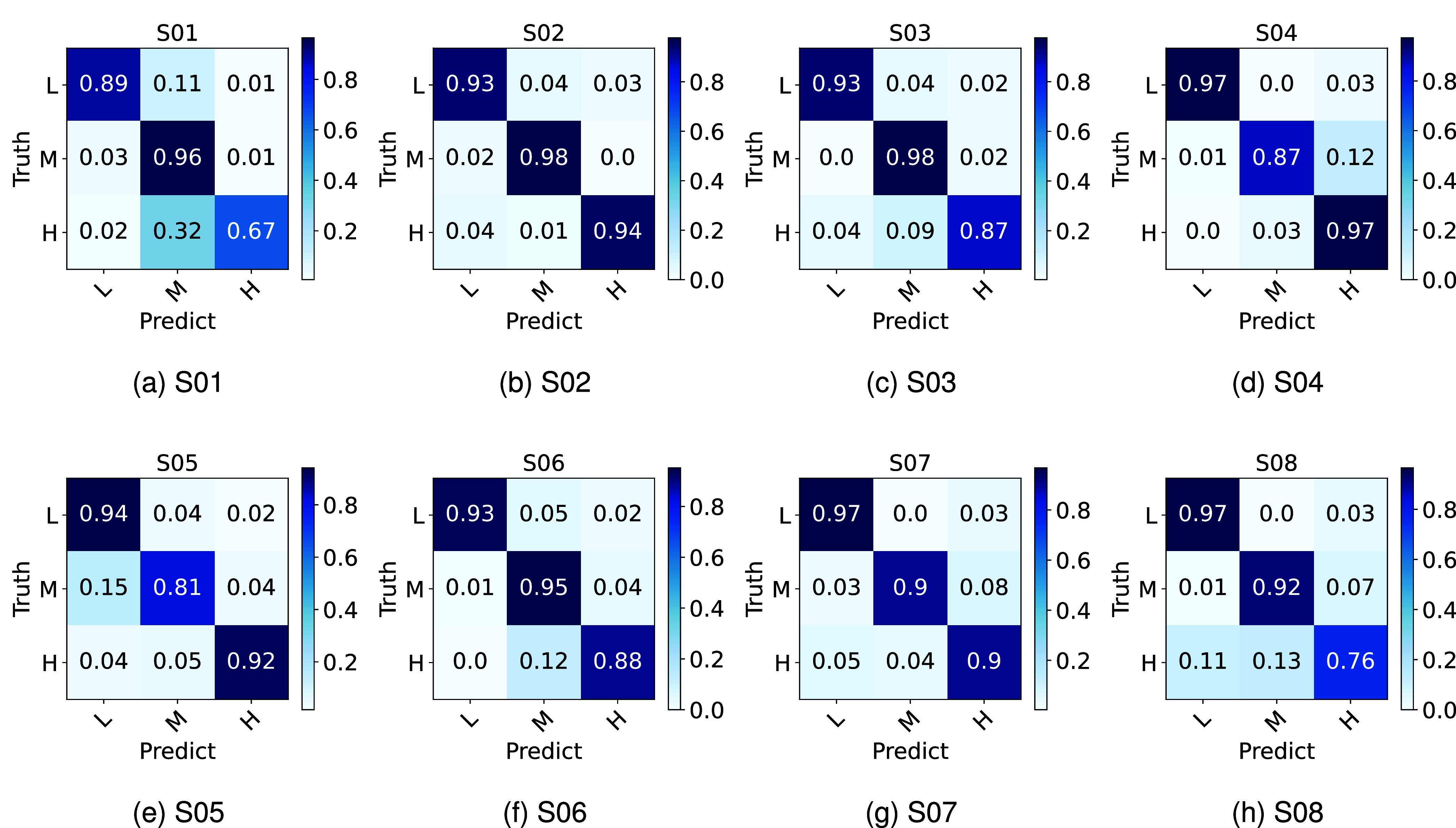
Confusion matrix for all subjects, (a) To (h) represent subjects 1-8, *L*, *M*, and *H* represent represent low workload, moderate workload, and high workload states, respectively.

The confusion matrices for each of the eight subjects are shown in Fig. 6. The evaluation accuracy for all workload states in the confusion matrices of subjects 2 and 7 exceeded 90%. Additionally, the accuracy for all workload states in the confusion matrices of subjects 3 to 6 was above 80%. The lower evaluation accuracy for subjects 1 and 8 under high workload conditions highlights the presence of individual differences.

**FIGURE 7.**
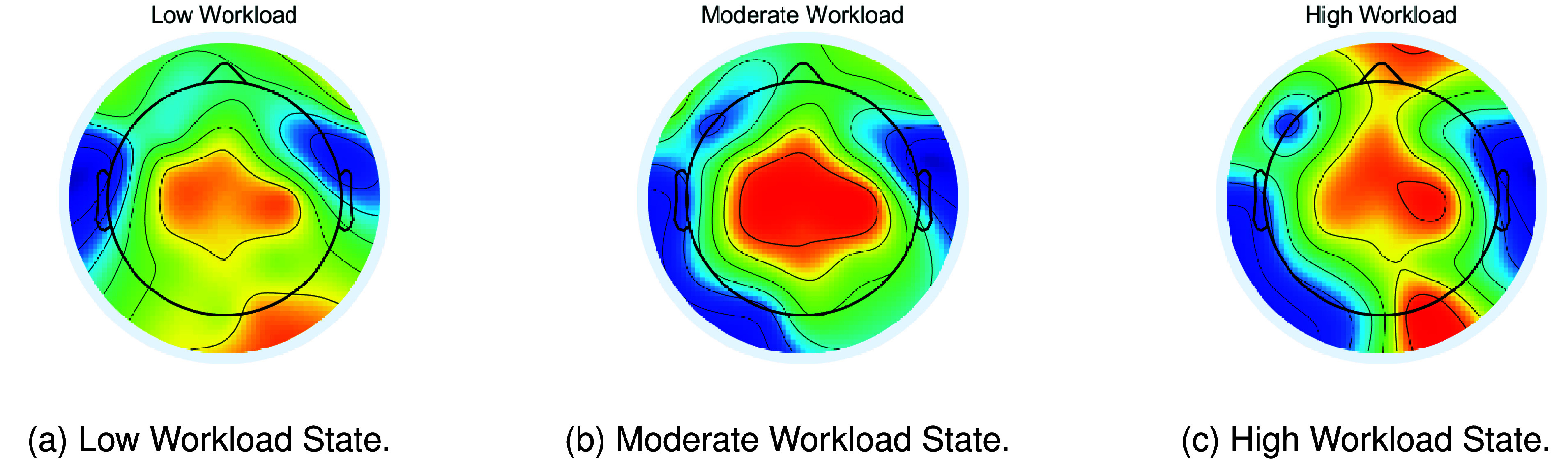
A representative pilot’s EEG topography maps.

To further demonstrate the performance of our proposed framework, visualization experiments were conducted on the collected dataset, as depicted in Fig. 7. For all workload states, the parietal lobe of the brain has a stronger activation response. Specifically, similar activation responses were observed in the parietal lobe for low workload states (shown in Fig. 7a) and high workload states (shown in Fig. 7c). However, under moderate workload conditions, the parietal lobe exhibits a stronger activation response (shown in Fig. 7b), which is not very similar to the other two states.

## IV. Discussion

Accurately identifying the workload status of pilots during flight is a critical challenge in civil aviation safety. In this work, we proposed a TRM-MAAN method that combined EEG and EMG to address the problem of pilot workload evaluation effectively. This method adopted a Transformer-based temporal relation modeling approach for effective feature extraction. Simultaneously, the adversarial training of the multi-modality feature fusion module and the modal classifier was applied to reduce distribution shifts between different modalities for better feature fusion. The effectiveness of TRM-MAAN has been verified by the improved accuracy of pilots’ workload evaluation, which has a positive effect on pilot safety in driving and in terms of pilot healthcare. The effectiveness of TRM-MAAN has been verified by the improved accuracy of pilots’ workload evaluation, which positively affects pilots’ healthcare. The proposed method also has potential value for clinically monitoring fatigue, psychological state, cognitive psychology, and other disorders.

This work attempts for the first time to use a multimodal fusion strategy based on Transformer and adversarial alignment for pilot workload evaluation. The key to achieving optimal performance is adjusting the network depth, especially the number of blocks in the multi-modality feature fusion module. The depth of the network is closely related to the dataset’s size and the task’s complexity. For example, in the field of vision, when the number of blocks in ConvNeXt is set to 18 or 36, the corresponding visual model achieves the best accuracy [40], [41]. However, our research results were inconsistent due to the relatively small dataset used for pilot workload evaluation. Therefore, we determined through experiments that three ConvNeXt blocks were appropriate. Increasing the model’s depth may overly emphasize the details of individual characteristics, leading to overfitting. Conversely, reducing the model’s depth may limit its learning ability and lead to underfitting. Fig. 3 illustrates the impact of block count on classification accuracy. Using different numbers of blocks can lead to performance degradation. It is worth noting that the model depth determined in our task can also guide similar functions that rely on small sample physiological signal-based pilot workload evaluation datasets.

Based on the results of Table 3, it can be seen that TRM-MAAN performs satisfactorily on all subjects, with an average accuracy of $91.90~\pm ~1.72\%$ ( $p < 0.05$). This indicates that the model is highly effective in evaluating workload and is highly competitive among all subjects. Furthermore, the evaluation performance of three multimodal models was compared, as shown in Fig. 4. Compared to the other two multimodal models (MHyEEG, Husformer), TRM-MAAN outperformed them in terms of evaluation accuracy for each subject, with improvements of 17% and 13% ( $p < 0.05$), respectively. Results in Figure 4 and Table 4 demonstrated the advantages of the proposed method, which can effectively reduce the inter-modal distribution bias while extracting complex temporal features by combining the temporal relationship modeling capability of the Transformer and the multimodal adversarial alignment strategy. In contrast, MHyEEG extracted features using an encoder consisting of multiple fully connected layers, which was not accurate enough in mining and portraying the temporal information and ignored the distributional differences between different modalities. Husformer used the cross-attention mechanism in the Transformer without considering aligning features from different modalities before feature fusion. Moreover, it concatenated features from different modalities along the temporal dimension to perform self-attention for context information aggregation, and the quadratic complexity of the self-attention operation resulted in significant computation costs.

Table 5 further demonstrated the superiority of TRM-MAAN in real-time performance evaluation. Specifically, TRM-MAAN can process each sample with lower latency and lower complexity while ensuring high accuracy, which is crucial for fast response in real-time systems. These results indicated that TRM-MAAN was not only competitive in terms of classification accuracy, but also capable of meeting the demands of real-time performance, paving the way for real-time monitoring of pilot driving safety and pilot health status.

The experimental results in this paper also verified the necessity of using various modules in TRM-MAAN, as shown in Fig. 5. Firstly, the experimental results highlighted the effectiveness of introducing the Transformer, with an average classification accuracy improvement of 5.75% compared to the MAAN method. This difference is due to the Transformer’s superior ability to process temporal data and capture long-range dependencies. It can better extract temporal features through self-attention mechanisms, reducing information loss and redundancy [42]. Similarly, compared to the TRM method, TRM-MAAN improved the average classification accuracy by 9.76%, demonstrating the effectiveness of adversarial alignment. The reason is that adversarial alignment can help the feature fusion of different modalities thereby enhancing the accuracy and reliability of overall recognition [43], [44].

It is worth noting that their respective characteristics determined the selection of EEG and EMG signals. EEG records brain electrical activity, providing real-time information about pilot attention, cognitive load, and fatigue status [45]. In addition, EMG measures muscle electrical activity, reflecting muscle tension and fatigue status during operation [46]. Therefore, combining the EEG and EMG signals offers the complementary advantage of providing real-time and direct pilot status, which can more effectively ensure flight safety and improve pilot operational performance. However, the EEG and EMG signals have different temporal characteristics and frequency band ranges. EMG exists in the frequency band of 20-2000 Hz with an amplitude of about 1-10 mV [47], whereas EEG exists in the range of 0.5-60 Hz with an amplitude of 10- $100~\mu V$ [48]. To address the challenge of fusing the two modalities, we extracted the features of EEG and EMG separately with a two-branch Transformer can effectively integrate the information of these two signals with different temporal features and frequency ranges. Then the features extracted from the two modalities were fused based on the data-driven adversarial alignment, which can help to better fuse features from different modalities, thus improving the overall recognition accuracy and reliability.

Experiments were conducted on each modality individually to demonstrate the effectiveness of the two modalities separately. The signals were input into a model containing a Transformer multi-modality feature extraction module and a ConvNeXt multi-modality feature fusion module, and comparative experiments were conducted with EEGNET, as shown in Fig. 5. Compared to EEGNET, the average accuracy of using EEG signals alone increased by 4.81%, reaching $89.75~\pm ~2.41\%$. This improvement indicates that although EEGNET has certain advantages in processing unimodal EEG signals, our proposed method, by combining the feature learning capabilities of Transformer and ConvNeXt, can more effectively capture essential information in EEG signals, thereby improving classification accuracy. In addition, experiments using EMG signals alone also demonstrated the advantages of our method. Although the improvement was not as significant as with EEG signals, it still showed some enhancement. This further validates the robustness and adaptability of TRM-MAAN in different modalities, laying the foundation for further improving the performance of multimodal fusion methods.

The confusion matrix in Fig. 6 reports the representative results of classifying three workload states of eight pilots using the TRM-MAAN method. It was found that, for subjects 1 and 8, some samples with high workload were misclassified as samples with moderate workload. Moreover, the proposed TRM-MAAN method correctly identified most samples of the other subjects, achieving a classification accuracy above 80% for all workload states. These results were partly attributable to the physiological variability of the pilots. Differences in physical conditions, physiological functions, and psychological states of different people can lead to considerable differences in physiological signals. Besides, we collected EEG and EMG signals during a long flight, during which possible loosening of electrode contacts and the effects of the pilot’s sweating affect the quality of the EEG and EMG signals, thus affecting the accuracy of the classification model. In addition, factors such as muscle strength and gesture posture change when pilots maneuver the joystick can also significantly impact the quality of EMG signals. Under the influence of these factors, subjects 1 and 8 demonstrated lower classification accuracy. However, by comparing the standard deviation of the classification accuracies of different methods across different subjects, we can find that the standard deviation of the accuracy of the proposed method (2.15%) was substantially reduced compared to other comparison methods (9.34% for MhyEEG and 3.87% for Husformer), which indicated that the proposed method showed a significant advantage in terms of robustness to overcome these influencing factors.

Research in biological psychology has demonstrated that the parietal lobes are critical in EEG emotion recognition experiments [49]. In a similar vein, our workload assessment experiment revealed that under consistent stimulation patterns (i.e., different stages of the flight task), the EEG topographic maps shown in Fig. 7 exhibited heightened activation responses in the parietal lobes across different workload states. Comparable high activation responses were noted in the parietal lobe for both low and high workload states. However, a stronger activation response was evident in the parietal lobe under moderate workload conditions. These findings highlight the complex relationship between brain activation patterns and varying workload intensities. The distinct neural signatures associated with each workload state offer valuable insights into the underlying cognitive processes and stress levels experienced by pilots.

Although our method performed well in the experiments, some limitations must be considered: 1) This work validated the algorithm on a smaller scale dataset, and the performance on larger scale dataset remained to be explored. 2) This work captured signals from two modalities, EEG and EMG, to validate the advantages of multimodal fusion. Efficient fusion strategies for more multimodal data and their impact on the evaluated performance were yet to be investigated. 3) External factors, such as electrode dislodgement or pilot sweating, affected the signal quality during the experiment, affecting the model performance. In addition, individual variability limited the generalization and usability of the model. Based on the limitations of this study, our future research will focus on the following aspects: 1) Expanding the number of subjects to validate the applicability and performance of the algorithm on larger datasets; 2) Adding more modalities to make effective use of the information of different modalities to improve the assessment accuracy; 3) Analyzing in-depth the potential factors that lead to the differences in the performances of different subjects, to further improve the model’s ability to adapt to different disturbances and even different individuals, to prompt the clinical application of the model. By addressing these issues, we hope to improve the robustness and versatility of the proposed method, thus laying a solid foundation for constructing a more reliable and accurate workload evaluation system suitable for clinical environments.

## V. Conclusion

In this paper, a novel TRM-MAAN framework is presented for pilots’ workload evaluation by using Transformer for temporal relationship modeling and adversarial alignment for multi-modal data fusion. The proposed TRM-MAAN framework outperforms common multimodal methods $(p< 0.05)$. The experimental results demonstrate the potential of the proposed TRM-MAAN framework to improve the accuracy and robustness of pilot workload assessment. By effectively integrating multimodal physiological signals, it can more accurately identify the workload of pilots, providing a promising method for improving flight safety. In this paper, a novel TRM-MAAN framework is presented for pilots’ workload evaluation by using Transformer for temporal relationship modeling and adversarial alignment for multi-modal data fusion. The proposed TRM-MAAN framework outperforms common multimodal methods (p<0.05). The experimental results demonstrate the potential of the proposed TRM-MAAN framework to improve the accuracy and robustness of pilot workload assessment. By effectively integrating multimodal physiological signals, it can more accurately identify the workload of pilots, providing a promising method for improving flight safety. However, this study is limited to a small dataset and inadequately explored individual performance differences due to possible external factors such as electrode detachment and individual differences. In the future, we will expand the size of the dataset and further investigate the modeling of robustness to external disturbances. In addition, effective cross-subject workload evaluation algorithms are further explored. All these works can pave the way for improving the usability and generalization of workload evaluation algorithms for better pilot safety and healthcare.
